# Muscle Oxygenation During Hypoxic Exercise in Children and Adults

**DOI:** 10.3389/fphys.2019.01385

**Published:** 2019-11-07

**Authors:** Anton Ušaj, Igor B. Mekjavic, Jernej Kapus, Adam C. McDonnell, Polona Jaki Mekjavic, Tadej Debevec

**Affiliations:** ^1^Faculty of Sport, University of Ljubljana, Ljubljana, Slovenia; ^2^Department of Automation, Biocybernetics and Robotics, Jozef Stefan Institute, Ljubljana, Slovenia; ^3^Department of Biomedical Physiology and Kinesiology, Simon Fraser University, Burnaby, BC, Canada; ^4^University Medical Centre Ljubljana, Ljubljana, Slovenia

**Keywords:** children, submaximal exercise, hypoxemia, total hemoglobin, deoxygenated hemoglobin

## Abstract

**Introduction:**

While hypoxia is known to decrease peak oxygen uptake ($\dot{V}\,o$_2 max_) and maximal power output in both adults and children its influence on submaximal exercise cardiorespiratory and, especially, muscle oxygenation responses remains unclear.

**Methods:**

Eight pre-pubertal boys (age = 8 ± 2 years.; body mass (BM) = 29 ± 7 kg) and seven adult males (age = 39 ± 4 years.; BM = 80 ± 8 kg) underwent graded exercise tests in both normoxic (P_i_O_2_ = 134 ± 0.4 mmHg) and hypoxic (P_i_O_2_ = 105 ± 0.6 mmHg) condition. Continuous breath-by-breath gas exchange and near infrared spectroscopy measurements, to assess the vastus lateralis oxygenation, were performed during both tests. The gas exchange threshold (GET) and muscle oxygenation thresholds were subsequently determined for both groups in both conditions.

**Results:**

In both groups, hypoxia did not significantly alter either GET or the corresponding $\dot{V}\,o$_2_ at GET. In adults, higher $\dot{V}$_E_ levels were observed in hypoxia (45 ± 6 l/min) compared to normoxia (36 ± 6 l/min, *p* < 0.05) at intensities above GET. In contrast, in children both the hypoxic $\dot{V}$_E_ and $\dot{V}\,o$_2_ responses were significantly greater than those observed in normoxia only at intensities below GET (*p* < 0.01 for $\dot{V}$_E_ and *p* < 0.05 for $\dot{V}\,o$_2_). Higher exercise-related heart rate (HR) levels in hypoxia, compared to normoxia, were only noted in adults (*p* < 0.01). Interestingly, hypoxia *per se* did not influence the muscle oxygenation thresholds during exercise in neither group. However, and in contrast to adults, the children exhibited significantly higher total hemoglobin concentration during hypoxic as compared to normoxic exercise (tHb) at lower exercise intensities (30 and 60 W, *p* = 0.01).

**Conclusion:**

These results suggest that in adults, hypoxia augments exercise ventilation at intensities above GET and might also maintain muscle blood oxygenation via increased HR. On the other hand, children exhibit a greater change of muscle blood perfusion, oxygen uptake as well as ventilation at exercise intensities below GET.

## Introduction

Exposure of humans to hypoxic environment provokes systemic hypoxemia that can compromise muscle oxygen availability (Richardson et al., 2006) and subsequently muscle function (Hoppeler and Vogt, 2001). Early compensatory physiological responses, including increased ventilation ($\dot{V}$_E_) and heart rate (HR) aim to counteract the reduced O_2_ availability and thereby augment systemic O_2_ saturation (SpO_2_) (Kriemler et al., 2016) and blood flow (Wolfel and Levine, 2001), respectively. Increased perfusion of organs or tissues, such as skeletal muscle, is also a well-established mechanism that can alleviate local hypoxaemia (Wolfel and Levine, 2001; Joyner and Casey, 2014). While these responses have previously been documented in both adults and children (Kriemler et al., 2016), full compensation is only achieved after prolonged acclimatization and only at moderate altitude/hypoxic levels (West et al., 2007). Furthermore, the aforementioned mechanisms do not seem to acutely counteract the hypoxia-related decrement in maximal endurance performance as this is mostly underlined by decreased $\dot{V}\,o$_2__max_ (Roach and Kayser, 2001; Astrand et al., 2003). Nevertheless, they may importantly modulate responses to submaximal exercise, which is even more pertinent, as both children and adults most often engage in submaximal activities at elevated altitudes (e.g., family mountaineering, skiing and/or trekking).

Physiological responses to hypoxic exercise are modulated by both the exercise intensity and the hypoxic stimulus. While the responses of adults and children to exercise in moderate hypoxia (Fraction of inspired O_2_ (F_i_O_2_) = 0.15) seem comparable (Springer et al., 1991), two important child-specific factors need to be considered when interpreting the hypoxia-related $\dot{V}$_E_ regulation. In particular, the sensitivity of peripheral receptors seems to be higher in children as compared to adults (greater carotid bodies sensitivity), and hypoxic exercise-related end tidal partial pressure of CO_2_ (P_ET_CO_2_) is lower in children than adults (Springer et al., 1989). Hypothetically, the enhanced sensitivity of the chemoreceptors should increase the $\dot{V}$_E_ response, but the hypocapnia, that occurs during hypoxic exercise tends to blunt the $\dot{V}$_E_. Furthermore, exercising in a hypoxic environment profoundly augments the cardiovascular response (Wolfel and Levine, 2001). Importantly, one of the key responses – to maintain adequate oxygen flux to exercising muscles – is the augmentation of systemic blood flow and consequently exercise-related hyperaemia (i.e., increased muscle blood volume) (Wolfel and Levine, 2001). While this phenomenon has been extensively studied in clinical and healthy adult populations (Joyner and Casey, 2015) no study to-date has scrutinized these responses in children.

Finally, it is also important to note that children do seem to have a different motor unit recruitment pattern (Dotan et al., 2012) as well as a different muscular structure (Lexell et al., 1992; Sallum et al., 2013) compared to adults. Therefore, the purpose of this study was to assess whether muscle oxygenation responses to hypoxic exercise may differ between adults and children during graded submaximal exercise.

## Materials and Methods

### Participants

Physically active, healthy, near sea level residing (<500 m) boys (Tanner stage 1) and adult males were eligible to participate in the present study. Exclusion criteria included smoking (adults only), asthma and any hematological or kidney disorder. The recruited participants in both groups had the following baseline characteristics: Children (*n* = 8; age = 8 ± 2 years (mean ± SD); body mass = 29 ± 7 kg; stature = 136 ± 11 cm; BMI = 16.0 ± 1.1 kg.m^–2^; body fat = 10.8 ± 5.6%); Adults (*n* = 7; age = 39 ± 4 years; body mass = 80 ± 8 kg; stature = 181 ± 5 cm; BMI = 24.3 ± 1.2 kg.m^–2^; body fat = 16.9 ± 7.7%). All participants were informed regarding the aims and potential risks of the study and provided written informed consent prior to their inclusion in the study. Additional informed consent was also obtained for all children from their parents. All adults were fathers of the participating children. The study was approved by the National Medical Ethics Committee of Slovenia and performed according to the guidelines of the Declaration of Helsinki.

### Study Outline

The present study was part of a bigger research project investigating potential differential effects of exercise and hypoxia on various physiological responses in adults and children. A detailed outline of the project along with some of the results has been published previously (Kapus et al., 2017). The participants arrived at the experimental facility, situated at an altitude of 940 m (Planica Olympic centre, Rateče, Slovenia) 1 day prior to the start of the tests. All participant underwent a familiarization incremental cyclo-ergometry protocol during the day prior to experimental testing in order to exclude potential learning and habituation effect. Advanced arrival also enabled appropriate nutritional and activity standardization before the tests. The participants were not allowed to consume any caffeinated (or alcohol-containing for adults) drinks during the 24-h pre-test period. All experimental protocols were performed in a laboratory environment under controlled, thermoneutral conditions (ambient temperature = 21.7 ± 1.8°C; relative humidity = 43 ± 4%).

### Experimental Procedures

The body fat content of the participants was assessed using skin fold measurements and calculated via the Jackson and Pollock (1978) equation. On the 1st day, the participants performed a submaximal incremental load exercise on an electrically braked cycle ergometer (Ergo Bike Premium, Daum electronics, Fürth, Germany) in normoxia (NOR). The testing protocol consisted of a 2-min resting period, followed by a 3-min warm up at 30 W. Subsequently the workload was increased by 30 W every 3 min. The employed increments, albeit step-wise slightly high for the younger participants, were chosen in order to provide time for stabilization of physiological responses at respective power outputs and enable us to use the same protocol in both, children and adults. The overall test workload increase over time is in line with previous work in pediatric population (Mahon et al., 1998; Paridon et al., 2006). The participants were required to maintain a pedaling cadence of 60–70 rpm throughout the duration of the test. As the exercise intensity and power output increased, the test was terminated, if the participants were unable to sustain the prescribed pedaling cadence. Upon completion of the exercise testing, and after dinner, the participants were confined to one floor of the hypoxic facility, which was continuously maintained normobaric hypoxic by reducing the F_i_O_2_ to 0.162 ± 0.003 [P_i_O_2_ (partial pressure of inspired O_2_) = 105 ± 0.6 mmHg; simulated altitude of ∼3,000 m]. The participants slept in normobaric hypoxic conditions (HYPs), and remained exposed to the hypoxic environment throughout the following day. The exercise protocol described above was then repeated in the HYP. Collectively, the participants were thus continuously exposed to normobaric HYP for approximately 12 h prior to the start of the HYP exercise tests.

Continuous breath-by-breath gas exchange was recorded throughout the testing protocol using a metabolic cart (Quark CPET, Cosmed, Rome, Italy) connected to a facemask (Vmask, 7500 series, Hans Rudolph Inc., Shawnee, OK, United States). Prior to each test the O_2_ and CO_2_ sensors of metabolic cart were calibrated using two calibration gas mixtures (CO_2_ fraction: 5% and O_2_ fraction:16%) along with the flowmeter calibration in line with the manufacturer’s instructions. HR and SpO_2_ were measured continuously using a fingertip pulse oximetry device 3100 WristOx (Nonin Medicals, Plymouth, MN, United States). Near infrared spectroscopy (NIRS; Oxymon MK III, Artinis Medical systems, Zatten, Netherlands) was employed to assess the relative changes in oxygenated hemoglobin (O_2_Hb) and deoxygenated hemoglobin (HHb) concentrations. Total hemoglobin (tHb), an index of tissue perfusion, was calculated from the O_2_Hb and HHb values. The NIRS probes were positioned over the (distal) belly of the right vastus lateralis muscle. Ink marks applied to the skin ensured that the probes were positioned at the same site in both NOR and HYP tests. The median fat layer above the measurement location, assessed using a calibrated skin fold caliper, was comparable between the two cohorts (Adults = 14 ± 6 mm; Children = 16 ± 3 mm; *p* = 0.12).

The basic principle and theory of NIRS is detailed elsewhere (Ferrari et al., 2004). Briefly, the laser light of 760 nm is emitted through the skin and absorbed by the hemoglobin. The magnitude of the absorption of near infra-red light is dependent on the oxygen bound to the hemoglobin (Hb), specifically if Hb is saturated with O_2_ (oxyhaemoglobin; O_2_Hb) or not (deoxyhemoglobin; HHb). A portion of the light is then reflected back to the optical detector. The signal is then analyzed to obtain values of O_2_Hb, HHb and total hemoglobin content (tHb). The data were continuously recorded and calculated at 50 Hz frequency. Although it is assumed that alterations in O_2_Hb and HHb reflect changes in muscle $\dot{\text{V}}\,\text{o}$_2_, any concomitant alterations of muscle blood flow and perfusion must also be considered. Acceptable reproducibility of the muscle deoxygenation measurement using the NIRS technique in children has previously been demonstrated (Leclair et al., 2010). Recently, it was recognized that the continuous wave NIRS technique is very sensitive to the thickness of the subcutaneous fat layer, which may illicit errors in the results of muscle oxygenation (Van Beekvelt et al., 2001; Niemeijer et al., 2017). In order to avoid these issues two approaches were employed: (a) relative values were used, by dividing all values with the resting average value; (b) comparisons between different participants (different thickness of fat layers) were avoided. Only within-subject comparisons within each group separately in normoxic and HYPs were performed.

### Data Analysis and Statistics

All statistical analyses were performed using the SPSS package (version 15.0, SPSS Inc., Chicago, IL, United States) and Sygma Plot 11 (Systat, San Hose, CA, United States). Data are presented as means ± standard deviations throughout the manuscript. The *a priori* set alpha level of significance was 0.05. The data were initially tested for normality of distribution using the criteria of skewness or kurtosis. Significant differences between both environmental conditions and exercise intensity were performed using paired *t*-test and ANOVA for repeated measurements for adults and children separately. *A posteriori* statistical power analysis for the main outcomes indicated that for all statistically significant comparisons and parameters with significant main effects or interactions a power of ≥0.80 was achieved (*N* = 7 and α = 0.05).

The muscle oxygenation and cardiovascular data were averaged for the last 30-s of each interval at designated exercise power outputs, and the values obtained from the NOR and HYP trials were compared using a two-tailed paired t-test.

**FIGURE 1:**
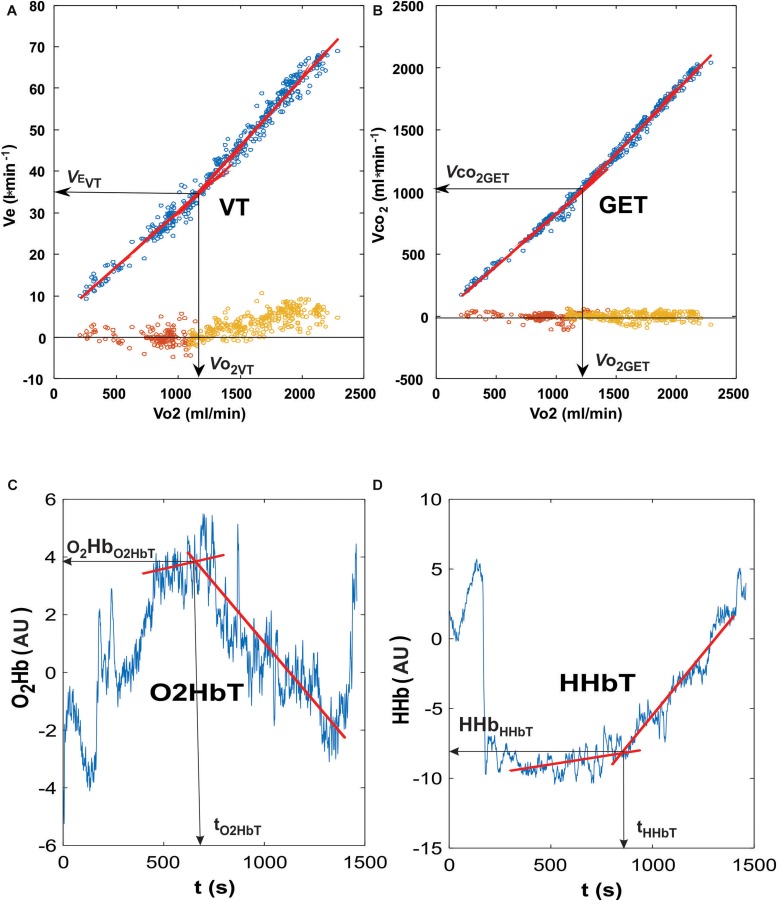
Representative curves for the ventilatory and oxygenation thresholds used in the present analysis. The determination of Ventilatory Threshold (VT; **A)** was performed using the intersection point of the two best fitting lines (the lowest residuals at the bottom of the diagram, around zero line) when plotting minute ventilation and oxygen consumption. The gas exchange threshold (GET; **B)** was determined by using the intersection point of the two best fitting lines when plotting carbon dioxide and oxygen consumption. Residuals were plotted at the bottom of the graph (around zero axis value). Both the oxygenated hemoglobin (O_2_Hb) and deoxygenated hemoglobin (HHb) thresholds **(C,D**, respectively) represent the intersection points of the two best fitted lines of measured values.

During the course of the exercise protocol, minute ventilation ($\dot{V}$_E_), CO_2_ output ($\dot{V}\,c\,o$_2_) and all three parameters of muscle oxygenation (O_2_Hb, HHb, and tHb) demonstrated a two-phase response: an initial gentle alteration followed by a steeper enhancement during the latter stages of the exercise. We calculated the intersection point of the two best $\dot{V}$_E_ fitted regression lines in the low and steep parts of $\dot{V}$_E_ vs. $\dot{V}\,o$_2_ diagrams using MATLAB R2015b (Mathworks Inc., Natick, MA, United States) for detecting the Ventilatory Threshold (VT) (Figure 1; Beaver et al., 1986). Using the same method, we also determined the Gas Exchange Threshold (GET), where $\dot{V}\,c\,o$_2_ values were analyzed in relation to $\dot{V}\,o$_2_ (Beaver et al., 1986). Due to the fact that also O_2_Hb, HHb and tHb follow the similar patterns (Grassi et al., 1999; van der Zwaard et al., 2016), temporal thresholds were also determined from the three NIRS-derived values (O_2_HbT, HHbT, and tHbT, respectively) (Figure 1). In addition to the time interval (t) from beginning of the exercise to the moment of occurrence of a particular threshold (T), the following relative parameters were also calculated: $\dot{V}$_E_ at VT ($\dot{V}$_EVT_), $\dot{V}\,o$_2_ at GET ($\dot{V}\,o$_2GET_), HR at GET (HR_GET_), $\dot{V}\,c\,o$_2_ at GET ($\dot{V}$_*co*__2GET_), O_2_Hb at O_2_HbT (O_2_Hb_O__2__HbT_), HHb at HHbT (HHb_HHbT_), and tHb at tHbT (tHb_tHbT_) (Figure 1). Pearson’s correlation coefficients were calculated between ΔtHb and ΔHR values (calculated differences between normoxic and HYPs for tHb and HR, respectively) at each workload (power output).

## Results

The nocturnal exposure to normobaric hypoxia resulted in a significant reduction in SpO_2_ and P_ET_O_2_ in adults and children during the resting period immediately prior to the HYP exercise tests (*p* < 0.01; Table 1). The resting P_ET_CO_2_ values were comparable between conditions. Interestingly, the pre-exercise HR was significantly increased only in adults (*p* = 0.02), albeit a tendency for an increase in children (Table 1). Absolute as well as body mass-normalized ventilatory and gas exchange parameters were not significantly higher in HYP in neither group (Table 1).

**TABLE 1:** Pre-exercise resting values of the measured cardiorespiratory parameters in adults and children.

	**Adults**			**Children**		
	**NOR**	**HYP**	***p***	**NOR**	**HYP**	***p***
$\dot{V}$_E_ (l min^–1^)	14 ± 2	16 ± 2	0.25	9 ± 4	11 ± 4	0.12
$\dot{V}$_E_ (l min^–1^⋅kg^–1^)	0.18 ± 0.04	0.20 ± 0.03	0.25	0.28 ± 0.06	0.32 ± 0.08	0.12
$\dot{V}\,o$_2_ (ml⋅min^–1^)	473 ± 72	496 ± 66	0.62	272 ± 58	338 ± 106	0.05
$\dot{V}\,o$_2_ (ml⋅min^–1^⋅kg^–1^)	6.0 ± 1.1	6.2 ± 0.7	0.69	8.9 ± 1.8	10.6 ± 2.3	0.07
$\dot{V}\,c\,o$_2_ (ml⋅min^–1^)	393 ± 73	426 ± 45	0.45	252 ± 62	290 ± 89	0.15
$\dot{V}\,c\,o$_2_ (ml⋅min^–1^⋅kg^–1^)	5.0 ± 1.2	5.3 ± 0.5	0.50	7.6 ± 1.6	8.7 ± 2.2	0.15
HR (1 min^–1^)	70 ± 7	76 ± 4	0.02	102 ± 18	106 ± 12	0.22
SpO_2_ (%)	96 ± 1	91 ± 2	< 0.01	96 ± 1	91 ± 2	< 0.01
P_ET_O_2_ (kPa)	13.1 ± 1	9.3 ± 0.6	< 0.01	13.6 ± 0.9	9.6 ± 0.7	< 0.01
P_E__T_CO_2_ (kPa)	4.1 ± 0.8	4.2 ± 0.3	0.75	3.9 ± 0.7	3.9 ± 0.2	0.79

*$\dot{V}$_*E*_: minute ventilation; $\dot{V}\,o$_2_: oxygen uptake; $\dot{V}\,c\,o$_2_: carbon dioxide output; HR: heart rate; SpO_2_: capillary oxygen saturation; P_*ET*_*O*_2_: end-tidal oxygen tension; P_*ET*_*CO*_2_: end-tidal carbon dioxide tension.*

All adults and children, except for one child, finished all of the required stages needed for the assessment of the submaximal muscle oxygenation and cardio-respiratory responses reported. Even though one child was unable to finish the third (90 W) stage under HYP we were still able to calculate the values of the main parameters required for the final analysis.

**FIGURE 2:**
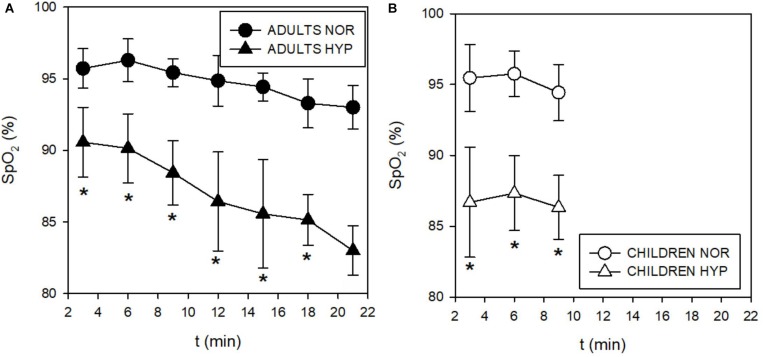
Time course of changes in capillary oxygen saturation (SpO_2_) during incremental test in adults **(A)** and children **(B)**. The SpO_2_ decreased in both adults and children during hypoxic conditions.

**FIGURE 3:**
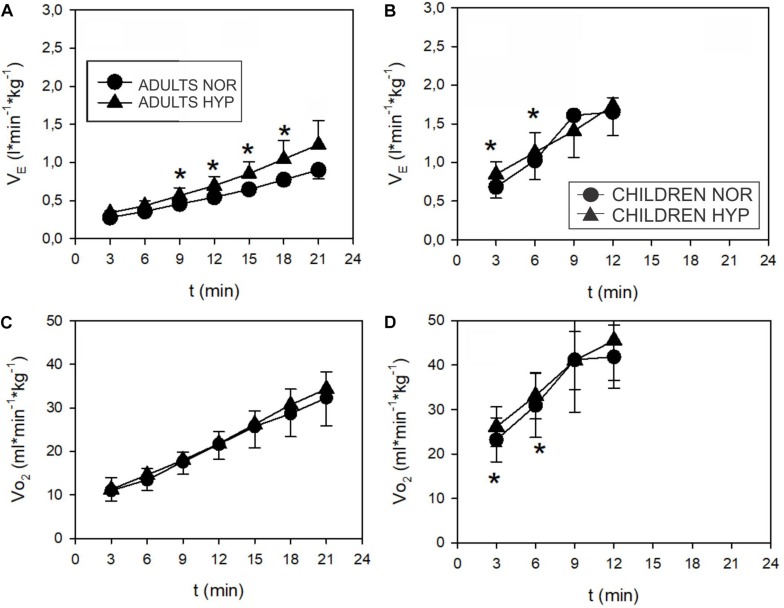
Time course of minute ventilation ($\dot{V}$_E_) and oxygen uptake ($\dot{V}\,o$_2_) (values normalized to body mass) during incremental test in adults **(A,C)** and children **(B,D)**. Adults increased $\dot{V}$_E_ more (^∗^*p* < 0.05) in hypoxia (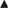) than in normoxia (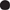). The difference starts above VT. $\dot{V}\,o$_2_ didn’t show any influence of hypoxia in adults. Children increased their $\dot{V}$_E_ and $\dot{V}\,o$_2_ at 3 and 6 min (below VT) (^∗^*p* < 0.05) in hypoxia (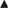) more than in normoxia (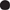).

**FIGURE 4:**
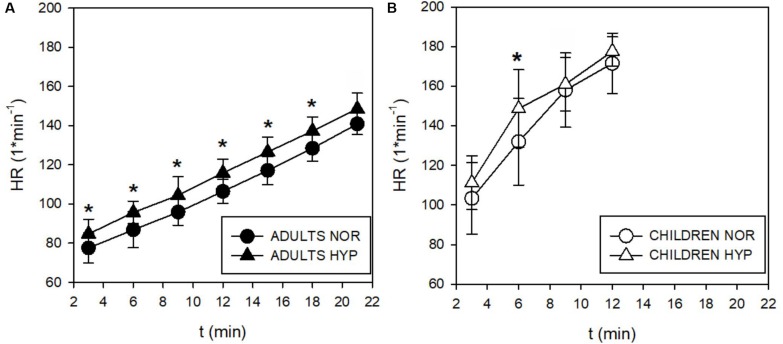
Time course of the heart rate (HR) responses during incremental test in adults **(A)** and children **(B)**. The HR increased by 11 beats⋅min^–1^ throughout the whole test (^∗^*p* < 0.05). Children didn’t enhance their HR in spite of a tendency for increases observed below their GET at 3 and 6 min in hypoxia (Δ).

During the incremental exercise test the SpO_2_ was significantly lower in the HYP compared to the NOR in both adults and children (Figure 2). Similarly, hypoxia significantly increased $\dot{V}$_E_ during exercise in both, adults (Figure 3A), and in children (Figure 3B). While adults $\dot{V}$_E_ was augmented during exercise intensities above VT and GET, children showed larger differences at lower intensities below VT and GET (Figure 3B). Whereas hypoxia did not significantly influence the exercise $\dot{V}\,o$_2_ response in adults (Figure 3C), it led to a significant elevation in children (*p* < 0.05) at intensities (30 and 60 W) below VT and GET (Figure 3D). Furthermore, hypoxia did not influence $\dot{V}\,c\,o$_2_ values during incremental test in any group. The anticipated increase in HR with increasing workload (Figure 4) was similar in adults and children, with hypoxia augmenting the response only in adults (*p* = 0.02) throughout the whole protocol, while a significant difference (*p* < 0.05) was noted in children only at intensities close to or below GET (Figure 4).

**FIGURE 5:**
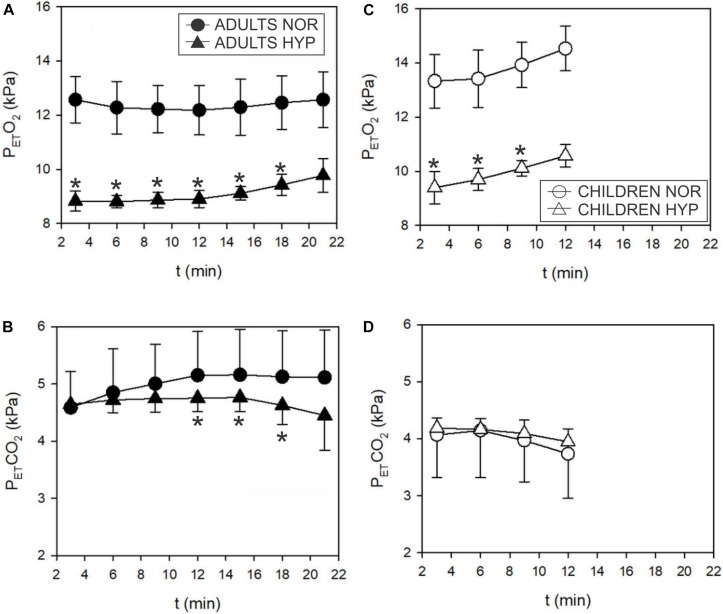
Time course of the end tidal partial pressure of O_2_ (P_ET_O_2_) and CO_2_ (P_ET_CO_2_) responses during incremental test in adults **(A,B)** and children **(C,D)**. P_ET_O_2_ decreased in hypoxia both, in adults (▲) and children (Δ). P_ET_CO_2_ decreased in hypoxia (▲; ^∗^*p* < 0.05) above GET only in adults. Hypoxia didn’t seem to influence P_ET_CO_2_ in children.

Exercise values of P_ET_O_2_ were lower in the HYP trial for both adults and children (Figures 5A,B). The decrease in P_ET_CO_2_ in adults at higher intensities (Figure 5C) was concomitant with the increase in $\dot{V}$_E_ (Figure 3A). In contrast to the adults, the increase in $\dot{V}$_E_ at lower intensities in children (Figure 3B) was not reflected in changes of P_ET_CO_2_ (Figure 5D). These values were already low in children in both NOR and HYP conditions (Figure 5D).

**FIGURE 6:**
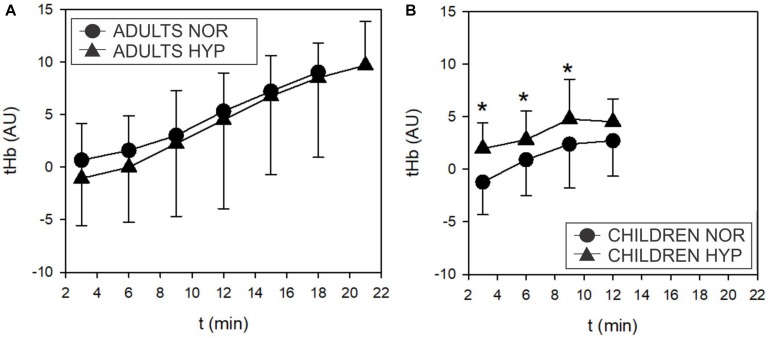
Time course of the changes in total muscle hemoglobin (tHb) concentrations during the incremental test in adults **(A)** and children **(B)**. Adults didn’t show any influence of hypoxia on tHb. However, children displayed higher tHb values in hypoxia compared to normoxia (Δ; *F* = 7.618, *p* = 0.01).

O_2_Hb and HHb were not altered during HYP exposure in adults and children, however, tHb exhibited higher values (*F* = 7.618; *p* = 0.01) at 30, 60 and 90 W in children in the HYP trial (Figure 6) but not in adults.

No significant differences were noted at similar relative exercise intensities represented by the threshold values (t_vt_, t_GET_, t_O__2__Hb_T, t_HHb_T, and t_Hb_T) between NOR and HYP conditions in either adults or children (Table 2). With the exception of higher HR_VT_ in adults and HR_GET_ observed in children (Table 3), hypoxia did not influence any of the cardiac or muscle oxygenation threshold parameters. Also, no hypoxia-induced differences in the ventilatory and GET parameters were noted both, when the threshold data was calculated as a function of the absolute values or when normalized to participants body mass (Table 3).

**TABLE 2:** Ventilatory and gas-exchange thresholds presented as time intervals from beginning of exercise to the moment of occurrence of particular threshold obtained during the graded cycling exercise test in adults and children.

	**Adults**			**Children**		
	**NOR**	**HYP**	***p***	**NOR**	**HYP**	***p***
t_VT_ (s)	720 ± 161	694 ± 198	0.84	368 ± 123	297 ± 152	0.23
t_GET_ (s)	625 ± 162	630 ± 85	0.82	308 ± 122	293 ± 145	0.78
t_O2Hb_ (s)	641 ± 227	659 ± 216	0.89	368 ± 182	350 ± 140	0.75
t_HHb_ (s)	635 ± 303	628 ± 209	0.96	387 ± 158	355 ± 98	0.54

*VT: ventilatory threshold; GET: gas exchange threshold; O_2_Hb: oxygenated hemoglobin; HHb: de-oxygenated hemoglobin.*

**TABLE 3:** Values of ventilatory, gas exchange and muscle oxygenation thresholds during the graded cycling exercise test in adults and children.

	**Adults**			**Children**		
	**NOR**	**HYP**	***p***	**NOR**	**HYP**	***p***
$\dot{V}$_E VT_ (l min^–1^)	37 ± 8	45 ± 12	0.27	25 ± 6	25 ± 13	0.90
$\dot{V}$_E VT_ (l min^–1^⋅^–1^)	0.49 ± 0.14	0.56 ± 0.18	0.32	0.76 ± 0.18	0.73 ± 0.28	0.83
$\dot{V}$_E GET_ (l min^–1^)	34 ± 7	43 ± 7	0.07	23 ± 4	25 ± 11	0.56
$\dot{V}$_E GET_ (l min^–1^⋅kg^–1^)	0.45 ± 0.12	0.54 ± 0.10	0.07	0.72 ± 0.13	0.75 ± 0.22	0.76
$\dot{V}$_co2 VT_ (ml⋅min^–1^)	1319 ± 444	1463 ± 376	0.59	676 ± 148	692 ± 331	0.91
$\dot{V}$_co2 VT_ (ml⋅min^–1^⋅kg^–1^)	17 ± 6	18 ± 5	0.67	21 ± 7	20 ± 7	0.81
$\dot{V}$_co2 GET_ (ml⋅min^–1^)	1225 ± 462	1292 ± 168	0.72	600 ± 120	660 ± 305	0.62
$\dot{V}$_co2 GET_ (ml⋅min^–1^⋅kg^–1^)	16 ± 7	16 ± 3	0.82	18 ± 6	19 ± 7	0.86
$\dot{V}\,o$_2 VT_ (ml⋅min^–1^)	1507 ± 447	1632 ± 351	0.68	785 ± 170	798 ± 324	0.93
$\dot{V}\,o$_2 VT_ (ml⋅min^–1^⋅kg^–1^)	20 ± 7	20 ± 5	0.75	25 ± 8	24 ± 7	0.81
$\dot{V}\,o$_2 GET_ (ml⋅min^–1^)	1288 ± 437	1493 ± 148	0.30	695 ± 142	776 ± 319	0.56
$\dot{V}\,o$_2 GET_ (ml⋅min^–1^⋅kg^–1^)	17 ± 7	19 ± 3	0.38	22 ± 7	23 ± 7	0.78
HR _VT_ (1 min^–1^)	107 ± 14	121 ± 10	0.04	153 ± 11	154 ± 24	0.98
HR _GET_ (1 min^–1^)	102 ± 9	109 ± 7	0.28	139 ± 13	156 ± 13	0.03
O_2_Hb_O2HbT_ (AU)	2.45 ± 4.98	3.63 ± 4.62	0.70	−1.53 ± 3.06	−0.66 ± 2.35	0.55
HHb_HHbT_ (AU)	−1.22 ± 7.18	−1.42 ± 5.22	0.95	0.76 ± 2.12	2.96 ± 1.76	0.09
tHb_tHbT_ (AU)	3.53 ± 8.37	1.96 ± 5.99	0.72	−1.20 ± 2.73	2.43 ± 3.08	0.05

*$\dot{V}$_*E VT*_: minute ventilation at ventilatory threshold; $\dot{V}$_*E GET*_: minute ventilation at gas exchange threshold; $\dot{V}$_*co2 VT*_: carbon dioxide output at ventilatory threshold; $\dot{V}$_*co2 GET*_: carbon dioxide output at gas exchange threshold;$\dot{V}\,o$_2 VT_: oxygen uptake at ventilatory threshold; $\dot{V}\,o$_2 GET_: oxygen uptake at gas exchange threshold; HR _*VT*_: heart rate at ventilatory threshold; HR _*GET*_: heart rate at gas exchange threshold; O_2_Hb_*O2HbT*_: Oxygenated hemoglobin values at Oxygenated hemoglobin threshold; HHb_*HHbT*_: De-oxygenated hemoglobin at de-oxygenated hemoglobin threshold; tHb_*tHbT*_: Total hemoglobin at total hemoglobin threshold.*

**FIGURE 7:**
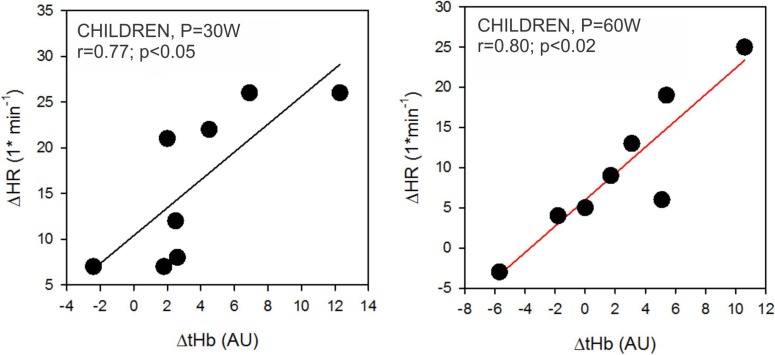
Correlations between hypoxia-related changes in heart rate (ΔHR) and total hemoglobin concentrations (ΔtHb) in children. Correlations were only significant at 30 W and 60 W power outputs as outlined in the left and right panel, respectively.

The hypoxia-induced increases of tHb (ΔtHb) and HR (ΔHR) for the same range of exercise intensities (up to 6 min of the incremental exercise, 30 and 60 W; below the GET and VT thresholds) correlated only in children (Figure 7). This relationship was not observed at higher intensities (longer than 9 min, 90 W). Furthermore, we did not observe any significant correlations between ΔtHb and ΔHR at 3, 6, and 9 min (30, 60, and 90 W) in adults.

## Discussion

The main finding of the present study is that, during hypoxic exercise changes in muscle oxygenation and select cardiorespiratory responses occurred at different exercise intensities and thresholds in adults and children. In particular, while children responded to hypoxic exercise by augmenting ventilation, oxygen uptake and muscle blood volume (perfusion distribution) at lower exercise intensities, below the corresponding thresholds, adults increased ventilation at higher intensities, above the VT and moreover increased HR, both during rest and throughout the entire exercise intensity range. Overall it seems that a comparable decrease in both SpO_2_ and P_ET_O_2_ were accompanied by different cardiovascular and respiratory modulations. In particular, the increased exercise HR in adults seems to enhance cardiac output, whereas in children a significantly less pronounced effect of hypoxia on HR was observed, albeit an enhanced muscle blood volume was noted. Also, as mentioned above, adults enhanced $\dot{V}$_E_ at higher exercise intensities only (above VT and GET), whereas children exhibited higher $\dot{V}$_E_ in hypoxia only at lower intensities (below their VT and GET).

Hypoxia-induced reductions in maximal endurance performance (Fulco et al., 1998; Roach and Kayser, 2001) and $\dot{V}\,o$_2max_ (Wehrlin and Hallen, 2006) are well documented. However, moderate hypoxia (F_i_O_2_ = 0.15) may also significantly influence certain characteristics during submaximal exercise, such as VT (Springer et al., 1991). While this reduction seems comparable in adults and children, the present results do not support the notion that VT is significantly decreased in either adults or children. In particular, our data, obtained at similar simulated altitude (≈ 3000 m) to that employed by Springer et al. (1991) indicate that such level of normobaric hypoxia (P_i_O_2_ = 105 ± 0.6 mmHg) does not significantly influence ventilatory (VT) or gas exchange (GET) thresholds. Also, the thresholds related to muscle oxygenation were not influenced by the utilized level of hypoxia. It, however, remains to be demonstrated if VTs might be altered as a consequence of exposure to greater hypoxia intensities (higher simulated or terrestrial altitude) and, moreover, if there is a threshold altitude above which significant differences between adults and children are observed. One possible reason for the aforementioned discrepancies could be differences in the exercise testing protocol characteristics. In particular, most studies to date, investigating children’s’ responses to exercise employed ramp style incremental tests (Mahon and Vaccaro, 1989; Springer et al., 1989, 1991). Thus, although the total increment in 3 min is identical in the protocols of previous and the current study, it has been achieved in 10 W/min steps in the previous studies, and in one 30 W step at the end of each 3-min period in the present one. According to the time-course of $\dot{V}\,o$_2_ observed during a 6-min steady state exercise at intensities below the GET, $\dot{V}\,o$_2_ kinetics in phase II (τ) are lower, but the amplitude of the increase remains unchanged in hypoxia (DeLorey et al., 2004). Meaning that $\dot{V}\,o$_2_ was different in HYP and NOR during the 1st min of exercise, but was similar for step increments between 2 and 6 min (DeLorey et al., 2004). Therefore, the difference in the step increments used in previous (10 W/min) (Boone et al., 2010; Zuniga et al., 2012; Jamnick et al., 2018) and present (30 W/3-min) study most likely resulted in different $\dot{V}\,o$_2_ kinetics, which may have contributed to the observed differences. Another possible explanation could be related to the differences in the hypoxic exposure duration. While Springer et al. (1991) executed their testing sessions immediately upon acute hypoxic exposure, the hypoxic sessions in the present study were conducted following a nocturnal continuous hypoxic exposure which might have altered exercise-related responses as a certain degree of, at least ventilatory adaptation, to hypoxia could already have been achieved (Debevec and Mekjavic, 2012).

With regards to exercise ventilatory responses, our results lend further support to previous studies showing augmented exercise $\dot{V}$_E_ during acute (Mekjavic et al., 1987) hypoxic exposure in adults, and furthermore indicate that children also exhibit augmented hypoxia-related ventilatory response (Springer et al., 1989, 1991). Nevertheless, the obtained data suggest that the two age groups demonstrate a slightly different temporal exercise related ventilatory responses to hypoxic stimulus. Namely, adults increased $\dot{V}$_E_ at intensities higher than VT and GET, while children had higher $\dot{V}$_E_ at intensities below these thresholds, therefore up to 6 min (30 and 60 W). At higher intensities children did not show any differences in $\dot{V}$_E_ between the NOR and HYP trials. This may be explained on the basis of the P_ET_O_2_ and P_ET_CO_2_ responses, and their relationship with $\dot{V}$_E_. While it has been reported that adults P_ET_CO_2_ is typically higher than in children at rest (Cooper et al., 1987), this was not noted in the present study. However, higher P_ET_CO_2_ levels in adults were noted at the lower intensities of exercise in present study. Similarly, $\dot{V}$_E_, P_ET_O_2_ and P_ET_CO_2_ also showed different response in adults and children. While P_ET_O_2_ increased throughout the exercise tests in children in NOR and HYP, it remained similar in both trials in adults. During exercise, P_ET_CO_2_ remained lower in children than in adults in NOR and HYP trials. Therefore, the P_ET_CO_2_ stimulus for enhancement of ventilation was lower in children than in adults. Consequently, the increased $\dot{V}$_E_ during the incremental exercise was higher in adults during higher intensities while children enhanced their $\dot{V}$_E_ only at lower exercise intensities.

As noted previously, the hypoxia-induced $\dot{V}$_E_ augmentation serves as a compensatory mechanism for alleviation of systemic hypoxemia (Springer et al., 1989; Smith et al., 2001). Interestingly, recent investigation on healthy adults (Chacaroun et al., 2019) only observed significant hypoxia-related $\dot{V}$_E_ augmentation during high but not sub-maximal intensity hypoxic trails. In addition, they reported no hypoxia-induced changes in NIRS derived muscle oxygenation index regardless of the intensity. In the present study, the augmented $\dot{V}$_E_ did not significantly influence SpO_2_ in adults, in the lower range of exercise intensities, since it was similar in the NOR and HYP trials. Therefore, the increased HR throughout the whole test at a rate of about 10–12 beats/min in HYP trials seemed to enhance blood flow in order to maintain similar oxygen flux to muscles (Joyner and Casey, 2014). However, because we were not able to detect significant enhancement of muscle O_2_Hb at unchanged $\dot{V}\,o$_2_, we speculate that, in adults, the O_2_ delivery by hypoxemic blood (reduced SpO_2_) may have matched its uptake in the HYP trial due to enhanced blood flow. Conversely, the children in the present study enhanced muscle blood perfusion distribution (muscle tHb) and also maintain or even enhanced $\dot{V}\,o$_2_ at lower exercise intensities despite the fact that HR was not significantly greater (HR increased only at one exercise intensity). The observed increase in oxygen uptake at the same exercise intensity during hypoxic vs. normoxic exercise in children seems somewhat intriguing given that previous work, albeit in trained individuals and performed in hypobaric hypoxia, indicated that submaximal oxygen uptake remains unaltered (Clark et al., 2007). However, factors such as hypoxia-related increase in the work of breathing (Sheel et al., 2010) as well as potentially compromised gross exercise efficiency (Noordhof et al., 2013) could have contributed to the observed $\dot{V}\,o$_2_, augmentation. In regards to the unaltered hypoxia-related HR responses our data are in contrast with other studies, where HR increases in response to hypoxia during exercise were consistently detected in children over a range of intensities (Springer et al., 1991). Nevertheless, our data collectively show that “compensatory vasodilatation” remains an important mechanism for the regulation of oxygen flux to the exercising muscles (Sarelius and Pohl, 2010; Casey and Joyner, 2011; Joyner and Casey, 2014).

In this regard it is important to emphasize that differences in muscle perfusion distribution between superficial and deep muscle layers during exercise have been previously reported (Okushima et al., 2015), this observation needs to be further explored. Especially since it currently remains unclear how blood perfusion is distributed among different muscle layers and different muscle fiber types. As demonstrated recently by Okushima et al. (2015) the studies employing traditional NIRS technology, that enables monitoring of the superficial muscle layers oxygenation provide limited understanding of the deep muscle perfusion and/or intermuscular oxygenation differences. In contrast, newer technology like the deep time resolved-NIRS instrument can differentiate between both layers. Also, NMR and PET scan techniques may provide further insight into the complex changes of muscle perfusion during exercise (Kalliokoski et al., 2006). In addition to the above-mentioned muscle layer perfusion differences, the reported dissimilarities in skeletal muscle fiber type distribution as well as capillarization could also, at least partly, explain the differential muscle oxygenation responses between adults and children. In particular, Sallum et al. (2013) previously demonstrated that both fiber size as well as capillary to fiber ratio in the quadriceps muscle are age dependent and tend to increase with age. Accordingly, lower capillary to fiber ratio in the children might therefore underlie greater blood perfusion variation in order to maintain adequate oxygen flux within the working muscles. Also, the reported differences in the skeletal muscle fiber type composition should be considered while interpreting the obtained results. It seems that maturation (from childhood to adulthood) provokes transformation of Type I to Type II fibers (Lexell et al., 1992) leading to higher percentage of Type II fibers in untrained adults compared to children. Indeed, trained adults seem to demonstrate similar muscle fiber type composition and total fiber number as prepubertal children (Ratel and Blazevich, 2017). Collectively, the above noted skeletal muscle morphological differences between children and adults could thus, result in dissimilar muscle penetration and subsequent sampling of the NIRS light (i.e., superficial layer in the adults and deeper layers in the children). Finally, even though the skinfold thicknesses under the NIRS probes measurement skin areas were comparable between adults and children potential differences in subcutaneous fat characteristics associated with aging (Weber et al., 2012) should also be taken into consideration.

### Limitations and Methodological Considerations

While the present study is the first to address the potential disparities in submaximal hypoxic exercise responses between adults and children and provides novel insight into muscle oxygenation modulation it is important to acknowledge a few limitations of the present work. First, the study employed a rather small sample size (*N* = 7 and 8) and was conducted in normobaric hypoxia. Thus, the obtained results should not be directly extrapolated to terrestrial (hypobaric) HYP (Faiss et al., 2013). In this regard, it is also of note that the control (normoxic) tests were performed in a slightly hypobaric condition (P_i_O_2_ = 134 ± 0.4 mmHg) due to the fact that the Planica facility is located at an altitude of 940 m. Second, the coordinated increase between muscle blood flow, influenced by HR and muscle blood perfusion distribution, estimated by tHb, may exist in children due to more homogenous recruitment of slow twitch motor units in children’s muscles below threshold levels (Dotan et al., 2012). In any case and due to the previously mentioned limitations of the NIRS technology, this hypothesis warrants further investigations preferably incorporating other experimental techniques to “directly” assess muscle blood flow [e.g., Doppler ultrasound or a similar approach still feasible and ethically acceptable in youth population; c.f. (Casey et al., 2008)]. Finally, to further expand our understanding of the submaximal responses to hypoxic exercise in adults and children it might be prudent to employ a constant-load submaximal exercise testing in both hypoxic and normoxic condition as opposed to the incremental load exercise employed in the current study.

## Conclusion

Even though the oxygenation and cardio-respiratory responses to maximal hypoxic exercise are quite well characterized, hypoxia-related alterations at submaximal exercise remain poorly investigated. Accordingly, the present study aimed to further our understanding of these phenomena. Collectively, our results suggest the following three specific response patterns to submaximal hypoxic exercise in adults and children: (a) HR seems to increase in adults across all intensities while it was only slightly increased at lower intensities in children; (b) the significant increase in ventilation was only observed at lower intensities in children and conversely, only at higher intensities in adults; and (c) muscle blood volume was only augmented in children at lower exercise intensities. The observed differential responses of adults and children during hypoxic submaximal exercise may be a consequence of disparate physiological adaptation to maintain O_2_ flux to the exercising muscles. The variability in responses to submaximal hypoxic exercise between adults and children seems to be different to that observed at maximal exercise intensities and warrants further investigation employing greater sample sizes and various age groups to elucidate the potential maturation-related effects.

## Ethics Statement

The study was approved by the National Medical Ethics Committee of Slovenia and performed according to the guidelines of the Declaration of Helsinki.

## Author Contributions

AU, IM, and TD contributed to the conception and design of the study. AU, IM, JK, AM, PJ, and TD conducted the experiments. AU and TD analyzed the data. AU drafted the manuscript. All authors contributed to the manuscript revision, read, and approved the final version.

## Conflict of Interest

The authors declare that the research was conducted in the absence of any commercial or financial relationships that could be construed as a potential conflict of interest.
